# Supplementary material for: In Situ TEM Observation of Electric Field-Directed Self-Assembly of PbS and PbSe Nanoparticles
Source: Nanomaterials (Basel). 2025 Aug 18;15(16):1275. doi: 10.3390/nano15161275 (PMC12388880; doi:10.3390/nano15161275)
Supplement: Supplementary file 1 [file nanomaterials-15-01275-s001.zip › nanomaterials-3590371-supplementary.pdf]

Supplementary Information

In Situ TEM Observation of Electric Field-Directed Self-Assembly of PbS and PbSe Nanoparticles

Iryna Zelenina[1], Harald Böttner[2], Marcus Schmidt[1], Yuri Grin[1], Paul Simon[1*]

[1]*Max-Planck-Institut für Chemische Physik fester Stoffe, Nöthnitzer Str. 40, 01187 Dresden, Germany*

[2] *retired from Fraunhofer Institut für Physikalische Messtechnik, Georges-Köhler-Allee 30, 79110 Freiburg, Germany*

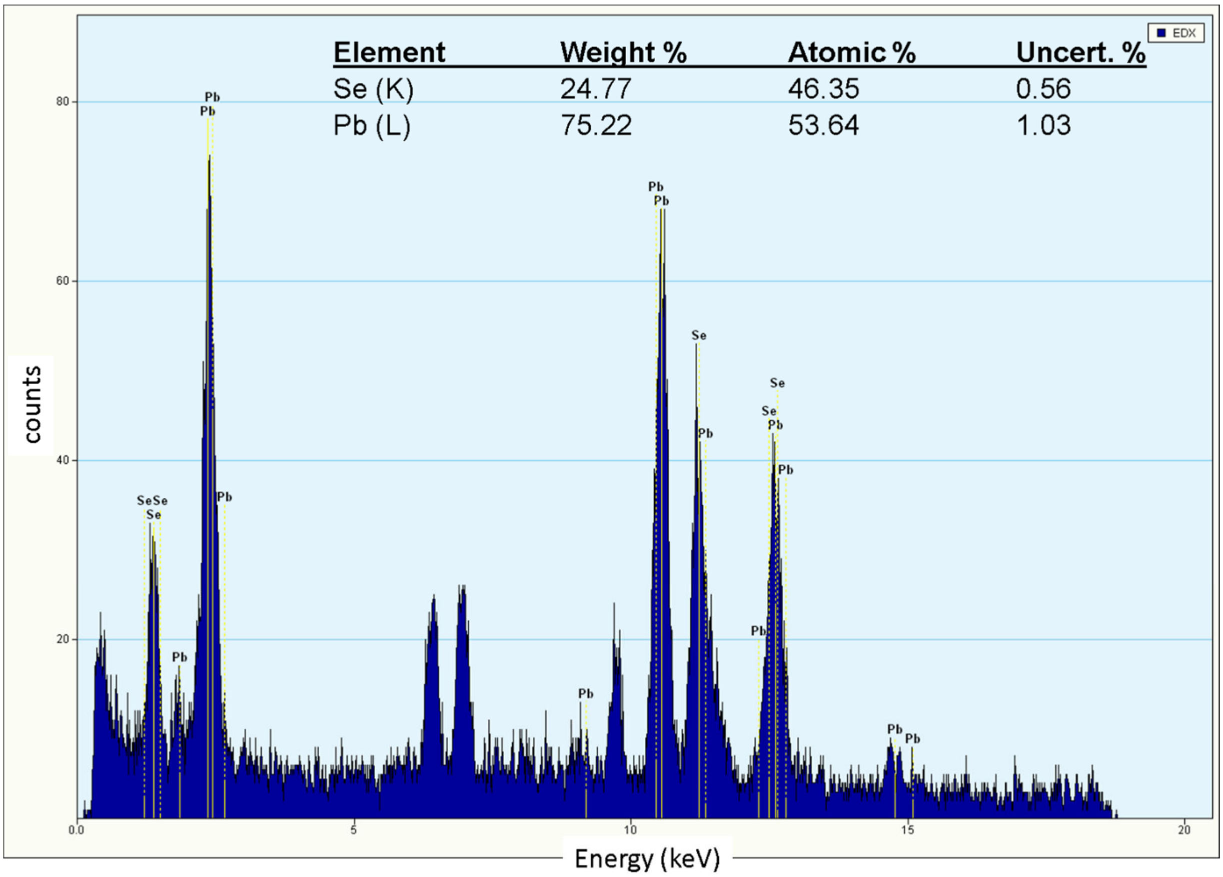

Supplementary Figure S1. EDX spectrum shows presence of 53.5 atomic % Pb and 46.3 atomic % Se.

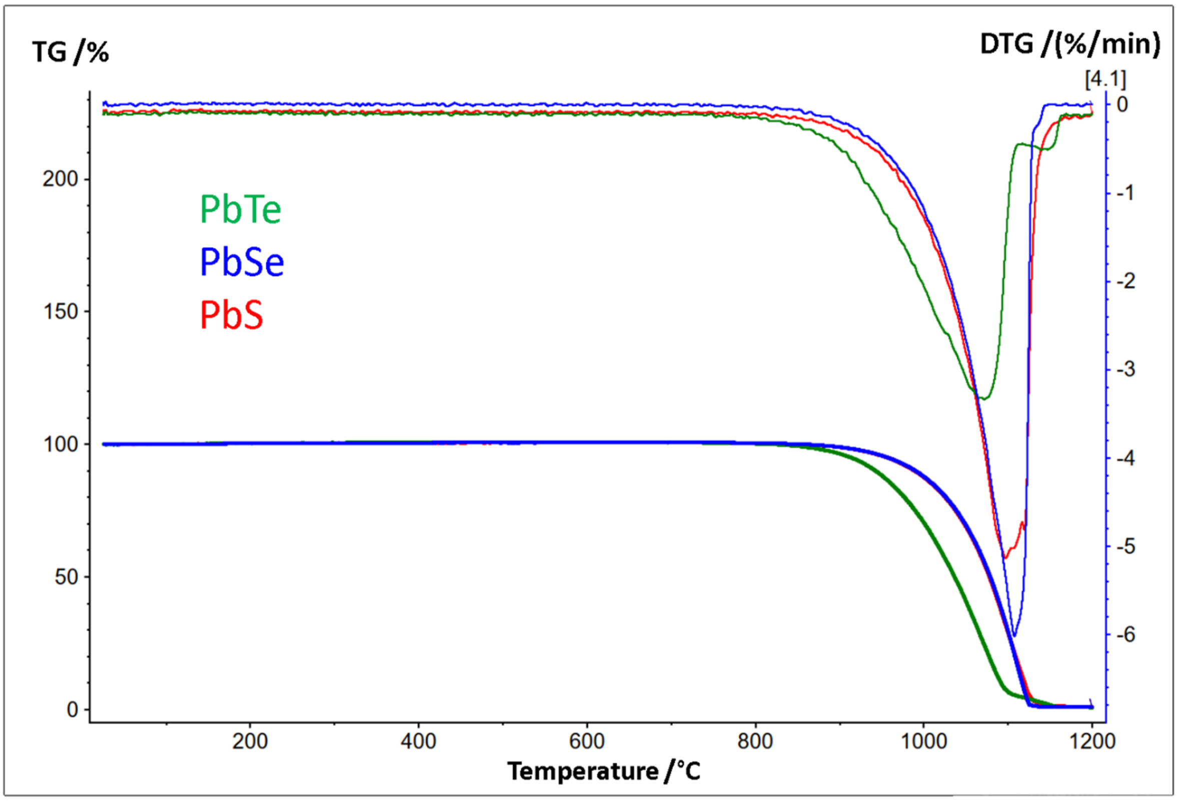

Supplementary Figure S2. Comparison of sublimation temperatures of lead chalcogenides under argon atmosphere. PbS and PbSe show very similar temperatures of 960 °C and 970°C (red, blue curves) and 925 °C for PbTe (green curve).

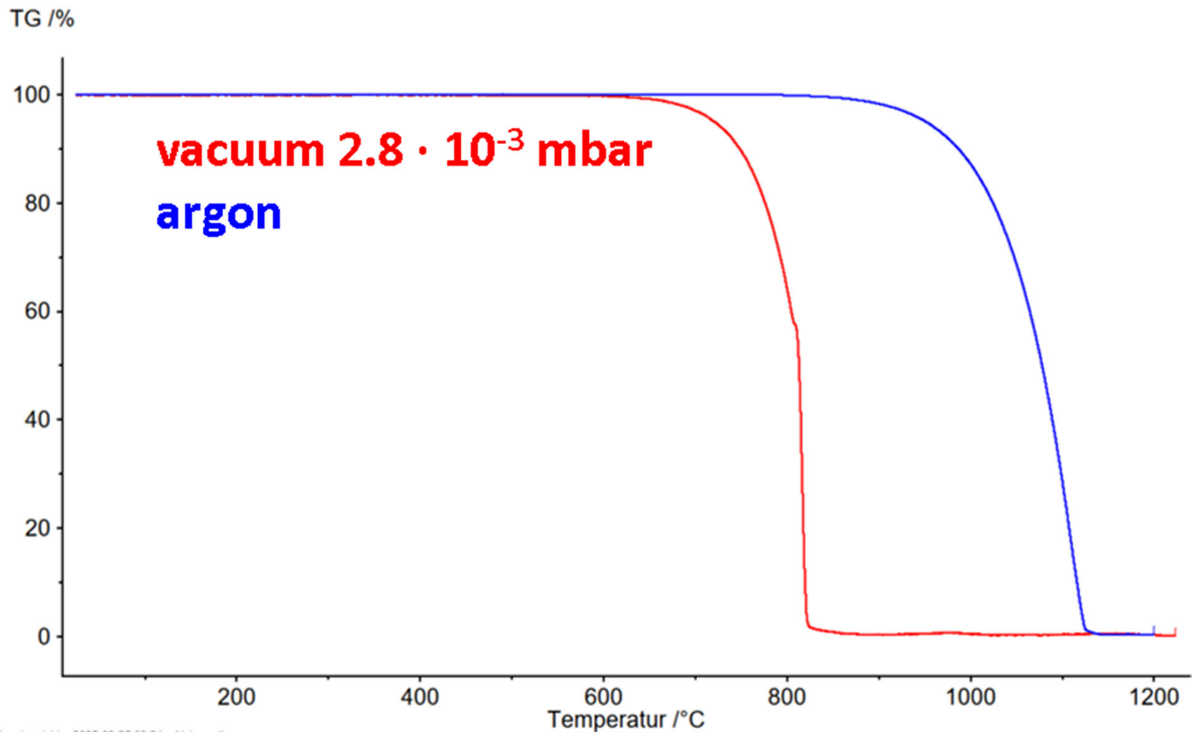

Supplementary Figure S3. Comparison of sublimation temperatures of PbSe under argon atmosphere and vacuum of 2.8×10^{-3} mbar measured with thermogravimetry. In argon at normal pressure, sublimation temperature of 960 °C (blue curve) is registered corresponding to onset of mass loss whereas for low vacuum (red curve) 660 °C is indicated. This corresponds to a shift of about 300 °C between the sublimation temperatures. Large mass lost in argon is observed above 1110 °C (blue) and for low vacuum (red) at about 800 °C given by the point of inflection.

Supplementary Table S1:

**Compound**	**Δ H°$_F$, 298 K** (kJ mol^{-1})
PbS	-98,3
PbSe	-100,0
PbTe	-68,6

Supplementary Table S1: Experimental enthalpies of formation of lead chalcogenides at 298K [1].

Supplementary Table S2:

**Compound**	**Δ H°$_F$, 0 K** eV/atom	**symmetry**
**PbO**	**-1.468**	**P4/nmm**
PbO	-1.240	Pca2$_1$
PbO	-1.251	Pbcm
**PbS**	**-0.784**	**Fm$\bar{3}$m**
PbS	-0.673	Pm3m
**PbSe**	**-0.561**	**Fm$\bar{3}$m**
PbSe	-0.487	Fmm2
**PbTe**	**-0.504**	**Fm$\bar{3}$m**
PbTe	-0.448	Pnma

Supplementary Table S2: Predicted enthalpies of formation of lead chalcogenides [2].

Supplementary Table S3:

**Compound**	**U exp** (kJ mol^{-1})	**U calc** (kJ mol^{-1})
PbO	3565	3332
PbS	3176	2918
PbSe	3138	2810
PbTe	-	2647

Supplementary Table S3: Lattice energies of lead chalcogenides [3].

Supplementary Table S4:

**Compound**	**Binding E** eV/molecule	**Binding E** **ionic**
PbS	32.6	30.5
PbSe	32.5	29.6
PbTe	31.4	28.1

Supplementary Table S4. Crystal binding energies of lead chalcogenides [4].

Supplementary Table S5:

**Compound**	**H sublim** (kJ mol^{-1})	**E cohesiv** (kJ mol^{-1})
PbS	235	2941
PbSe	227	2853
PbTe	219	2707

Supplementary Table S5. Crystal sublimation and cohesive energies of lead chalcogenides [5].

References

1. M. Binnewies, E. Milke, Thermochemical data of elements and compounds, 2*nd*, edition, Wiley-VCH, Weinheim, Germany **2002**.
2. https://legacy.materialsproject.org.
3. Oshchapovskii, V.V. Interpolation determination of the lattice energy of ionic crystals within the framework of stereoatomic model. Russ. J. General Chem., 2008, 78, 532–542. https://doi.org/10.1134/S1070363208040051.
4. H. Tanaka, A. Morita. The crystal binding energies of the IV–VI semiconductors. *J. Phys. Soc. Jpn*. **1979**, 46, 523-529.
5. K.K. Nanda. Paradox associated with cohesive energy of IV-VI semiconductors. *Phys. Lett. A*, **2020,** 384, 126645.
